# Consolidation electrochemotherapy with bleomycin in metastatic melanoma during treatment with dabrafenib

**DOI:** 10.2478/raon-2014-0035

**Published:** 2015-03-03

**Authors:** Sara Valpione, Luca G. Campana, Jacopo Pigozzo, Vanna Chiarion-Sileni

**Affiliations:** 1 Melanoma Oncology Unit, Veneto Region Oncology Research Institute (IOV-IRCCS), Padova, Italy; 2 Melanoma and Sarcoma Unit, Veneto Region Oncology Research Institute (IOV-IRCCS), Padova, Italy

**Keywords:** dabrafenib, electrochemotherapy, melanoma, BRAF inhibitors, bleomycin

## Abstract

**Background:**

Small molecules that inhibit V600 mutated BRAF protein, such as vemurafenib and dabrafenib, are effective in treatment of metastatic melanoma.

**Case report:**

We here describe the clinical course of a V600E BRAF mutated metastatic melanoma patient with systemic disease, who developed tumor progression on superficial soft-tissue metastases during treatment with dabrafenib. Bleomycin electrochemotherapy during dabrafenib treatment was administered to control the soft-tissue progressing metastases and ensured sustained local control without significant toxicity.

**Conclusions:**

The new combined approach maintained the patient quality of life and allowed for the prosecution of the target therapy, which proved to be still effective on systemic disease, up to 17 months.

## Background

In the last years, the treatment of V600 BRAF mutated metastatic melanoma patients has radically changed. Small molecules that inhibit V600 mutated BRAF protein, such as vemurafenib and dabrafenib, are now available and provide unprecedented response rates ranging to 50% 1,2, with median progression free survival of approximately 6 months. Further, the combination of BRAF inhibitors (BRAFi) and MEK inhibitors (*i.e*., trametinib or cobimetinib) ensured even more striking results, with response rates ranging to 70% and progression free-survival raising to approximately 10 months.^3^ The main toxicity related to BRAFi treatment is cutaneous, which, despite being not dose limiting in most cases, may impact on patient quality of life, that should instead be a priority in the context of the advanced phase of the disease.^4^

In order to further improve the outcome of patients with metastatic melanoma, researchers are exploring BRAFi in combination with other approaches. Unfortunately, the attempt of combining BRAFi with new immunoregolatory molecules such as anti-CTLA4 ipilimumab failed due to the increase of liver and skin toxicities^5^, and no data are available about combination with chemotherapy.

Another approach under active investigation is represented by the association of BRAFi with other locoregional treatments as radiotherapy and isolated limb perfusion. Although the best schedule of radiation and BRAFi remains to be established, this approach has shown an acceptable tolerability in preliminary experiences in patients with brain metastases.^6^ In this context, a sensitizing effect leading to enhanced cutaneous toxicity has been reported when BRAFi was concomitant with radiation and therefore should be carefully considered.^7^

Electrochemotherapy (ECT) is a combined locoregional treatment which has shown sustained activity in different tumor histotypes, including melanoma.^8^–^11^ The mechanism of action of ECT relies on the association of a poorly permeant anti-cancer agent (bleomycin or cisplatin) followed by locally-applied electric voltages which open transient pores on the cell membrane and increase drug entry into neoplastic tissues. Most common side effects of ECT include local erythema, skin ulceration and persistent pigmentation. This treatment permits a local complete response in 15–58% of patients with superficial metastases, according to the most recent experiences^9^,^12^,^13^ with ECT applied according to a shared protocol (European Standard Operative procedures of Electrochemotherapy, ESOPE).^10^

We here describe the clinical course of a meta-static melanoma patient who achieved durable tumor control by concomitant treatment with dabrafenib and bleomycin-ECT, administered to control isolated soft tissue metastases increasing during dabrafenib treatment.

## Case report

A 72-year-old metastatic melanoma male patient treated with dabrafenib 150 mg twice daily within the compassionate programme of Glaxo Smith Kline BRF115252. Bleomycin-ECT was administered in accordance with the ESOPE protocol. Treatment side effects were graded according to the Common Toxicity Criteria for Adverse Events (CTCAE), version 4. Written informed consent was obtained for the treatments and for the scientific use of clinical data, according to EC and Italian law requirements.

The patient received the first diagnosis of cutaneous melanoma of the left leg (stage IIA, T2b N0), in May 2007. From Dec 2010 to February 2011 he developed a single in-transit metastasis associated with inguinal lymph node recurrence, both managed by surgical excision. Thereafter, the patient was enrolled in an adjuvant trial with ipilimumab (CA184-029). Treatment was discontinued after four cycles owing to persistent G2 skin toxicity (pruritus), G2 diarrhoea and G2 hyphophysitis (he remained on therapy with corticosteroids for recurrence of hyphophysitis when he tried to discontinue them).

**FIGURE 1.**
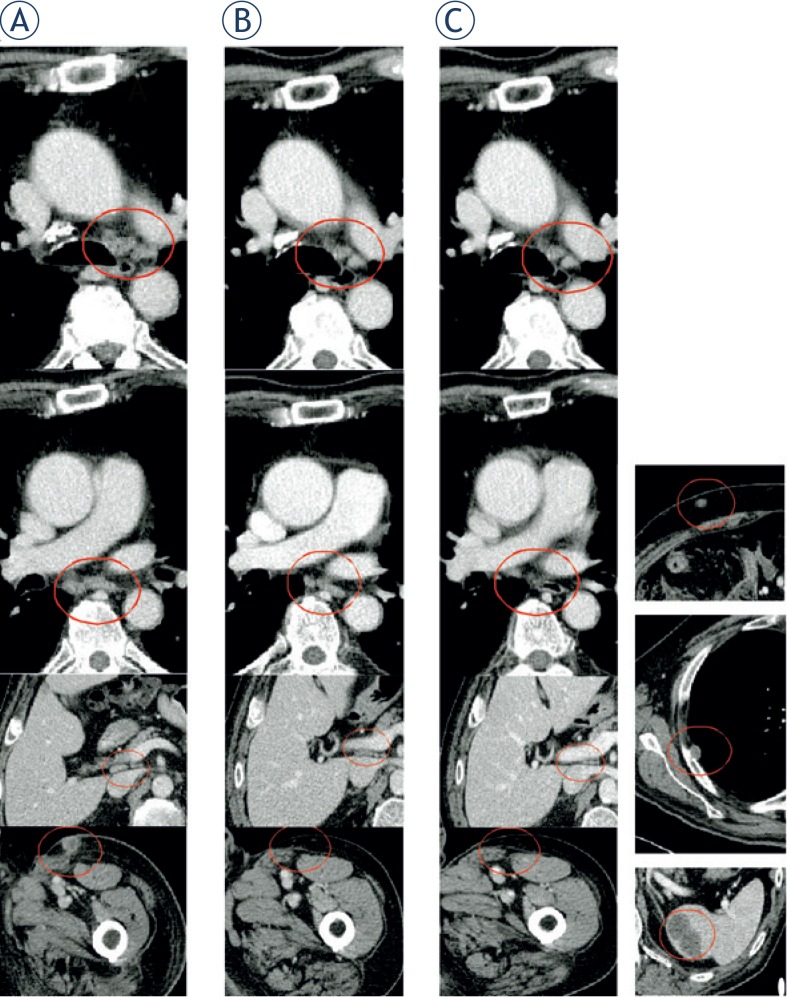
Radiological course of a metastatic melanoma patient initially treated with dabrafenib and subsequently temozolomide, combined with electrochemotherapy on superficial tumor nodules. **(A)** In July 2013 the patient had multiple nodal metastases; **(B)** in January 2013 was appreciated a good response to dabrafenib on visceral metastases (despite superficial soft tissue progression of the left leg); **(C)** in November 2013, despite the maintained control of the nodal metastases present at the beginning of dabrafenib treatment (left column), new deep (soft tissue, pleural and splenic, right column) metastases developed.

In May, two additional local recurrences were resected. In October 2011, temozolomide treatment was started for inoperable local recurrence. In May 2012, after an initial stabilization of the disease, he developed pathological mediastinal and abdominal lymph nodes (Figure 1A), and soft tissue progression in the left leg, rapidly increasing in size and number, requiring frequent dressing for bleeding and impairing the patient’s quality of life. Molecular analysis of BRAF gene was positive for V600E mutation. In June 2012, dabrafenib treatment (150mg per os twice daily, continuously) was started, obtaining in two weeks a partial response on superficial disease and complete response in mediastinal and abdominal lymph nodes (Figure 1B).

The treatment was well tolerated. In particular, despite previous skin toxicity during the adjuvant trial with ipilimumab, no significant cutaneous toxicity appeared during dabrafenib; the only reported adverse events were G1 plantar hyperkeratosis (from June 2012, controlled with acetylsalicylic acid based topical treatment) and a temporary G1 rhinitis (from October to December 2012).

**FIGURE 2.**
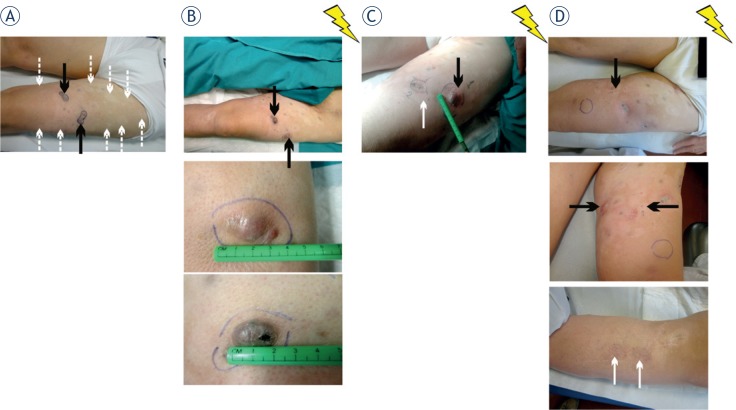
Clinical course of a metastatic melanoma patient initially treated with dabrafenib and subsequently temozolomide, combined with electrochemotherapy on superficial tumor nodules. In July 2013 the patient had multiple nodal and soft tissue metastases; **(A)** in October 2012 the left thigh of the patient was covered with white spots in the site of the metastases responsive to dabrafenib (white dotted arrows); at that time, only two bulging nodules were appreciable (black arrows); **(B)** despite the good response to dabrafenib on visceral metastases, in January 2013 three of these lesions worsened and one was ulcerated (black arrows); **(C)** in June 2013, after the first ECT course the metastases of the left thigh shrank; **(D)** in November 2013, new multiple skin metastases developed. Nevertheless, tumor control at the site of initial metastases that were responsive to dabrafenib and ECT was maintained (white arrows). Jagged arrows symbolize ECT courses.

In January 2013, the majority of subcutaneous lesions were stable except three, which progressively increased becoming symptomatic (Figure 2A and 2B).

Treatment with dabrafenib was maintained, considering the good tolerance and the systemic control of the disease, and ECT was applied in progressing nodules (the maximum diameter of the lesions was 3.5cm) under mild general sedation using systemic Bleomycin (15MUI).^14^,^15^. After one month, the lesions regressed to be classified as partial response (Figure 2C) without significant cutaneous toxicity; a second ECT cycle was administered in July 2013 without additive toxicity except for transient G1 erythema. Dabrafenib was stopped in November 2013 because of new soft tissue lesions and lymph node progression (Figure 1C and 2D). To assess whether a new clone with BRAF V600E mutation loss could have developed we performed a tumor biopsy, that confirmed the diagnosis of melanoma metastases and, interestingly, the maintenance of BRAF V600E mutation in the new soft tissue nodules. A third ECT cycle was applied with palliative intent in the new soft tissue lesions obtaining, again, a partial response on treated nodules; systemic therapy was switched to temozolomide, considering the previous efficacy and the impossibility to use immunomodulating therapy due to the corticosteroid need.

In March 2014, the patient is still mostly asymptomatic and fully-active with controlled disease and continues chemotherapy with temozolomide.

## Discussion

This is, to our knowledge, the first report of ECT combined with a BRAFi in a patient with metastatic melanoma. Actually, no consistent data are available about the prosecution of BRAFi treatment beyond progression in metastatic melanoma patients, but this case suggests that a multidisciplinary approach with locoregional treatments in addition to BRAFi therapy could be useful in some patients and should be tested more extensively. ECT seems a reliable and safe approach to control isolated skin and/or subcutaneous lesions in patients with systemic or visceral disease control. The combination of BRAFi and ECT allowed the control of disease and preservation of PS and quality of life for 17 months, which is almost three times the usual progression free survival time observed with the use of BRAFi.

This was a clear benefit for the patient, particularly in a disease like metastatic melanoma, where treatment options, despite the promising recent advances, are still numbered and long survival is difficult to obtain. The most important finding emerging from the presented case is the tolerability of the combined treatment approach. Radiotherapy is demonstrated to be effective in patients receiving BRAFi treatments, but a cautious evaluation, and interruption of the therapy during radiation, is recommended to avoid significant worsening of cutaneous toxicity.

We cannot elucidate whether the combination of dabrafenib and ECT had an additive effect, or whether ECT, thanks to its sustained local cytotoxic activity, may enhance tumor antigen presentation and promote lymphocyte tumor infiltration, as previously reported.^16^,^17^ Moreover, the immunological benefits could have been enhanced through the silencing of the aberrant immune-depressant pathways driven by the BRAF constitutive activation.^18^,^19^.

This may be a possible explanation for the benefit observed in this patient despite the multiple and rapid recurrences. Nevertheless, we cannot exclude that the disease course could be favored by the previous immunotherapy with ipilimumab.

In conclusion, we recommend further investigations to assess the following open issues: (a) whether the maintenance of BRAFi after tumor progression could give advantage to patients compared to its discontinuation when locoregional treatment, to control isolated tumor relapse, is possible; (b) to evaluate the most effective and tolerable locoregional treatment (*i.e.* radiotherapy, isolated limb perfusion/infusion, ECT).

In our experience, the association of ECT during dabrafenib proved to be a safe and valuable option in a challenging patient who developed tumor resistance exclusively on superficial disease. ECT ensured sustained local control on skin metastases without significant toxicity, maintained patient quality of life and allowed for the prosecution of target therapy which proved to be still effective on systemic disease. As a result, we encourage the study of this association in an adequate number of patients.
